# Programmed death-ligand 1 expression in carcinoma of unknown primary

**DOI:** 10.1186/s12885-024-12437-w

**Published:** 2024-06-06

**Authors:** Hye Min Kim, Ja Seung Koo

**Affiliations:** Department of Pathology, Yonsei University College of Medicine, Seoul, South Korea

**Keywords:** Carcinoma, Primary known, PD-L1

## Abstract

We examined the expression of programmed death-ligand 1 (PD-L1) in carcinoma of unknown primary (CUP) and its potential implications. Tissue microarrays were constructed for 72 CUP cases (histologic subtypes: 22 adenocarcinoma, 15 poorly differentiated carcinoma, 19 squamous cell carcinoma, and 14 undifferentiated carcinoma; clinical subtype: favorable type 17 [23.6%], unfavorable type 55 [76.4%]), with immunohistochemical staining performed for PD-L1 (22C3, SP142, SP263, and 28 − 8), CK7, and CK20 to determine the association between staining results and clinicopathological parameters. In CUP, the PD-L1 positivity rate was 5.6–48.6% (tumor cells [TC] or tumor proportion score [TPS]: 5.6–36.1%, immune cell score [IC]: 8.3–48.6%, combined positive score [CPS]: 16.7%) using different cutoff values for 22C3 (TPS ≥ 1%, CPS ≥ 10), SP142 (TC ≥ 50%, IC ≥ 10%), SP263, and 28 − 8 (TC and IC ≥ 1%). PD-L1 SP142 TC and PD-L1 SP263 IC showed the lowest (5.6%) and highest (48.6%) positivity rates, respectively. The PD-L1 positivity rate did not significantly differ based on the histologic subtype, clinical subtype, or CK7/CK20 across clones. Considering TC κ ≥ 1%, TC κ ≥ 50%, IC κ ≥ 1%, and IC κ ≥ 10%, the PD-L1 positivity rate was TC = 4.2–36.1% and IC = 9.7–48.6%; the overall agreement between antibodies ranged from 69.4 to 93.1%, showing fair or better agreement (κ ≥ 0.21). In CUP, PD-L1 positivity varied depending on antibodies and scoring systems, with no difference observed according to histologic or clinical subtypes.

**Supplementary Information:**

The online version contains supplementary material available at 10.1186/s12885-024-12437-w.

## Simple Summary

Carcinoma of unknown primary (CUP) refers to a heterogeneous collection of cancers where metastatic growth is observed, but the origin of the primary tumor remains unidentified. The type of primary cancer is critical for establishing the treatment strategy in metastatic carcinoma, presenting a considerable challenge in CUP. Patients with programmed death-ligand 1 (PD-L1)–positive tumors are well-known to benefit from targeted therapy against PD-L1. However, the expression of PD-L1 in CUP remains poorly explored. The present study demonstrated that PD-L1 was expressed in CUP with varying positivity rates depending on the antibody and scoring system employed. There was no difference in PD-L1 expression based on histological or clinical subtypes. Based on PD-L1 expression, immune checkpoint inhibitors could afford an effective treatment strategy in CUP.

## Introduction

Carcinoma of unknown primary origin (CUP) is a metastatic carcinoma in which the primary tumor remains elusive even after evaluation of the clinical history, physical examination, radiological findings, laboratory tests and other diagnostic investigations [1]. CUP accounts for approximately 5–15% of malignant tumors [2–4], and advances in imaging and molecular testing have reduced this proportion to 1–2% in recent years [5]. Histologically, CUP comprises adenocarcinomas (50–60%) or poorly differentiated carcinomas (30–40%), with other histological types, including squamous cell carcinomas (5–8%) and undifferentiated carcinomas (2–5%) [4, 6]. Although the precise nature of CUP remains uncertain, two main hypotheses have been suggested: the first postulates that CUP represents a true metastatic tumor with a primary focus that is markedly small to be identified; the second suggests that CUP is a distinct entity with independent characteristics due to regression or dormancy of the primary lesion, known as the ‘true’ or ‘true” genuine’ or ‘genuine’ CUP hypothesis [6].

Treatment planning for metastatic carcinoma is generally determined by the type of primary cancer, making the absence of a known primary tumor in CUP a critical treatment challenge. The traditional diagnostic and treatment algorithm for CUP involves identifying favorable subgroups by undertaking a traditional diagnostic work-up and administering tissue origin-specific therapy while administering empirical chemotherapy or tissue origin-specific therapy based on the characteristics of each CUP in unfavorable subgroups [7]. Techniques such as immunohistochemistry (IHC) and molecular tools such as gene expression profiling, miRNA expression, and DNA methylation analysis have been employed to determine the most appropriate tissue-of-origin for a specific CUP [8]. Furthermore, precision medicine concepts based on advances in genomic tools are being applied to CUP to attempt targeted therapy by identifying possible treatment targets [9]. Therefore, identifying an appropriate treatment target for CUP is crucial to ensure proper treatment.

Programmed death 1 (PD-1) is an immune checkpoint molecule found on different immune cells, playing a crucial role in immune responses [10]. Conversely, programmed death-ligand 1 (PD-L1) acts as a ligand for PD-1. Tumor cells express PD-L1, which facilitates their evasion of antitumor immune responses by interacting with PD-1 and forming a suppressive pathway [11, 12]. PD-L1 is expressed in 20–70% of tumors, including lung cancer [11, 13–16], urinary bladder cancer [17], malignant melanoma [18], ovarian cancer [19], breast cancer [20, 21], and gastric cancer [22, 23]. In patients with PD-L1-positive tumors, targeted therapy against PD-L1 can be used to induce an antitumor immune response. Notably, PD-L1 inhibitors have been approved as effective treatments for non-small cell lung cancer, urothelial carcinoma, gastric carcinoma, esophageal carcinoma, cervical cancer, and triple-negative breast cancer (TNBC) [24]. In addition, various drugs such as pembrolizumab, atezolizumab, durvalumab, nivolumab, and ipilimumab have been developed as PD-L1 inhibitors [25]. Therefore, it is important to determine whether PD-L1 is expressed in tumor cells prior to targeted therapy. The most common and simple method for detecting PD-L1 expression is IHC using a monoclonal PD-L1 antibody on formalin-fixed paraffin-embedded (FFPE) specimens. Monoclonal PD-L1 antibodies, such as clone 28 − 8 [26], 22C3 [27], SP142 [14, 17], and SP263 [28] are commercially available, and appropriate antibodies and scoring systems have been established as companion diagnostics for different types of cancer. Although several studies have investigated PD-L1 expression in various tumors using various antibodies, PD-L1 expression in CUP has been poorly explored. Therefore, the purpose of the present study was to examine PD-L1 expression in CUP according to different antibodies and scoring systems and to explore its implications.

## Materials and methods

### Patient selection and clinicopathologic evaluation

In this study, we utilized FFPE tissue samples obtained from patients with Carcinoma of Unknown Primary (CUP) at Severance Hospital. The study adhered to the principles of the Declaration of Helsinki and obtained approval from the Institutional Review Board of Yonsei University Severance Hospital (IRB number: 4-2022-1380). Due to the retrospective nature of the study, patient consent was exempted by the Institutional Review Board of Yonsei University Severance Hospital.

The selected patients were diagnosed with metastatic carcinoma by a pathologist between January 1999 and December 2012. In this study, needle biopsies yielding insufficient tissue for TMA construction were excluded, while excisional biopsies suitable for TMA construction were included. Cases that received chemotherapy or targeted therapy before tissue diagnosis were excluded. All available hematoxylin and eosin (H&E)-stained slides were carefully reviewed. Clinicopathological parameters, including patient age, sex, histological type, organ involvement, and patient outcomes, were assessed for each tumor. Based on histological criteria, CUPs were categorized into four distinct groups [29]: adenocarcinomas (ADCs) displayed glandular differentiation, while squamous cell carcinomas (SCCs) exhibited evidence of squamous differentiation. Poorly differentiated carcinomas (PDCs) did not exhibit any specific lineage differentiation, and undifferentiated carcinomas (UDCs) consisted of syncytial tumor cell nests or individual tumor cells closely intertwined with dense lymphoplasmacytic infiltration, resembling the pattern seen in nasopharyngeal undifferentiated carcinomas. Additionally, CUPs were classified into favorable and unfavorable subgroups according to international guidelines [7, 30]. In accordance with international guidelines, the following nine scenarios are defined as the favorable subgroup. In this study, these same nine scenarios were also defined as the favorable subgroup; (1) poorly differentiated neuroendocrine CUP, (2) well-differentiated neuroendocrine tumor of unknown primary, (3) peritoneal adenocarcinomatosis of a serous papillary in females, (4) isolated axillary nodal metastases in females, (5) SCC involving non-supraclavicular cervical lymph nodes, (6) CUP with a colorectal IHC or molecular profile, (7) single metastatic deposit from unknown primary, (8) males with blastic bone metastases or IHC/serum prostate-specific antigen expression, and (9) SCC involving isolated inguinal adenopathy. CUP cases outside the defined favorable subgroup were categorized as the unfavorable subgroup.

### Tissue microarray

Following the assessment of H&E-stained slides, suitable FFPE tumor tissue samples were retrospectively gathered, focusing on the most representative tumor region, which was carefully demarcated. A punch machine was utilized to extract the chosen area, and a 3 mm tissue core was inserted into a 6 × 5 recipient block. For each sample, tissue microarrays were created, with two tissue cores included in each array.

### IHC

Immunohistochemistry (IHC) was conducted on FFPE tissue sections, and the antibodies employed are specified in Supplementary Table 1. Briefly, 3-µm thick tissue sections were prepared from paraffin blocks and then deparaffinized and rehydrated using xylene and alcohol solution. The IHC procedure was carried out using a Ventana Discovery XT automated stainer (Ventana Medical System, Tucson, AZ, USA). Antigen retrieval was achieved using CC1 buffer (Cell Conditioning 1; citrate buffer, pH 6.0; Ventana Medical System). Immunohistochemical staining was performed, incorporating appropriate positive and negative controls. For the negative control group, the primary antibody incubation step was omitted. Each antibody’s recommended positive control, as specified by the manufacturer, was utilized.

### Interpretation of immunohistochemical results

Immunohistochemical staining of PD-L1 was performed according to the antibody used. PD-L1 22C3 expression was evaluated using tumor cells (TC) (tumor proportion score [TPS]), immune cell score (IC), and combined positive score (CPS). TPS was calculated by dividing the number of PD-L1 staining tumor cells by the number of viable tumor cells and multiplying by 100%. The CPS was calculated by dividing the number of PD-L1 staining cells (including tumor cells, lymphocytes, and histiocytes) by the number of viable tumor cells and multiplying by 100%. PD-L1 28 − 8, SP142, and SP263 were evaluated for TC and IC. TC was defined as the percentage of tumor cells showing any intensity of membranous staining for PD-L1, while IC was defined as the percentage of the tumor area occupied by PD-L1 staining immune cells (including lymphocytes, histiocytes, dendritic cells, and granulocytes). In this study, PD-L1 interpretation was conducted by two pathologists (HM Kim and JS Koo) who participated in the study, using a multi-view microscope. They determined TC, IC, and CPS of PD-L1 for each case while reviewing the TMA slides. For cases near the cut-off value, the two pathologists reached a final decision through consensus. The pathologist (JS Koo) who interpreted the PD-L1 IHC in this study is a board-certified pathologist with over 20 years of experience in the field. Their expertise lies particularly in breast cancer, where they have been routinely interpreting PD-L1 (SP142 and 22C3) for several years in daily practice. Additionally, they have published research papers on PD-L1 [31–33].

Two different methods were used to analyze the TPS, IC, and CPS. First, the cutoff values established for each PD-L1 clone in other tumor types were used. For PD-L1 22C3, TPS of ≥ 1 [34] and CPS of ≥ 10 were considered positive [35]. For PD-L1 SP142, TC of ≥ 50 and IC of ≥ 10 were considered positive [36]. For PD-L1 28 − 8 and SP263, TC and IC of ≥ 1 were considered positive [37]. Second, to compare the results for each antibody, the criteria for positivity were set as TC(TPS) ≥ 1%, TC(TPS) ≥ 50%, IC ≥ 1%, and IC ≥ 10%. For CK7 and CK20, the cutoff value was set at 10%; cases with < 10% staining were considered negative, whereas those with ≥ 10% staining were considered positive [38].

### Statistical analysis

Data analysis was performed using SPSS for Windows (version 24.0; IBM Corp., Armonk, NY, USA). Continuous variables were analyzed using Student’s t-test, while categorical variables were assessed using Fisher’s exact tests. The threshold for statistical significance was set at *p* < 0.05. To evaluate the agreement between any two PD-L1 antibody clones for each scoring method, Cohen’s kappa coefficient was utilized. The interpretation of the kappa coefficient values was as follows: <0 indicated no agreement, 0.0–0.20 represented slight agreement, 0.21–0.40 indicated fair agreement, 0.41–0.60 signified moderate agreement, 0.61–0.80 suggested substantial agreement, and 0.81–1.00 denoted almost perfect agreement [39]. Kaplan-Meier survival curves and log-rank statistics were employed to assess the survival time. Additionally, multivariate regression analysis was conducted using a Cox proportional hazards model.

## Results

### Basal characteristics of patients with CUP according to the histologic and clinical subtypes

Supplementary Tables 2 and 3 show the basal characteristics according to histological and clinical subtypes in the 72 CUP cases. Overall, 22 (30.6%) patients had ADC, 15 (20.8%) had PDC, 19 (26.4%) had SCC, and 16 (22.2%) had UDC. The clinical subtype was favorable in 17 (23.6%) and unfavorable in 55 (76.4%) cases. The involved organs were as follows: lymph nodes 49 (68.1%), bone 8 (11.1%), brain 7 (9.7%), and other 8 (11.1%). Moreover, there was a difference in clinical subtype according to the histologic subtype, with ADC and UDC showing a higher proportion of the unfavorable type, while SCC showed a higher proportion of the favorable type (*p* = 0.003). Additionally, postoperative treatment differed according to the histologic subtype, with chemotherapy most commonly employed in ADC, chemoradiotherapy in PDC, and surgery only in UDC (*p* = 0.007). Among the CUP cases, 37 (51.4%) were CK7 (+)/CK20 (-), 3 (4.2%) were CK7 (+)/CK20 (+), 3 (4.2%) were CK7 (-)/CK20 (+), and 29 (40.3%) were CK7 (-)/CK20 (-), with no significant difference in histologic subtype (*p* = 0.522).

### PD-L1 expression in CUP

**Fig. 1:**
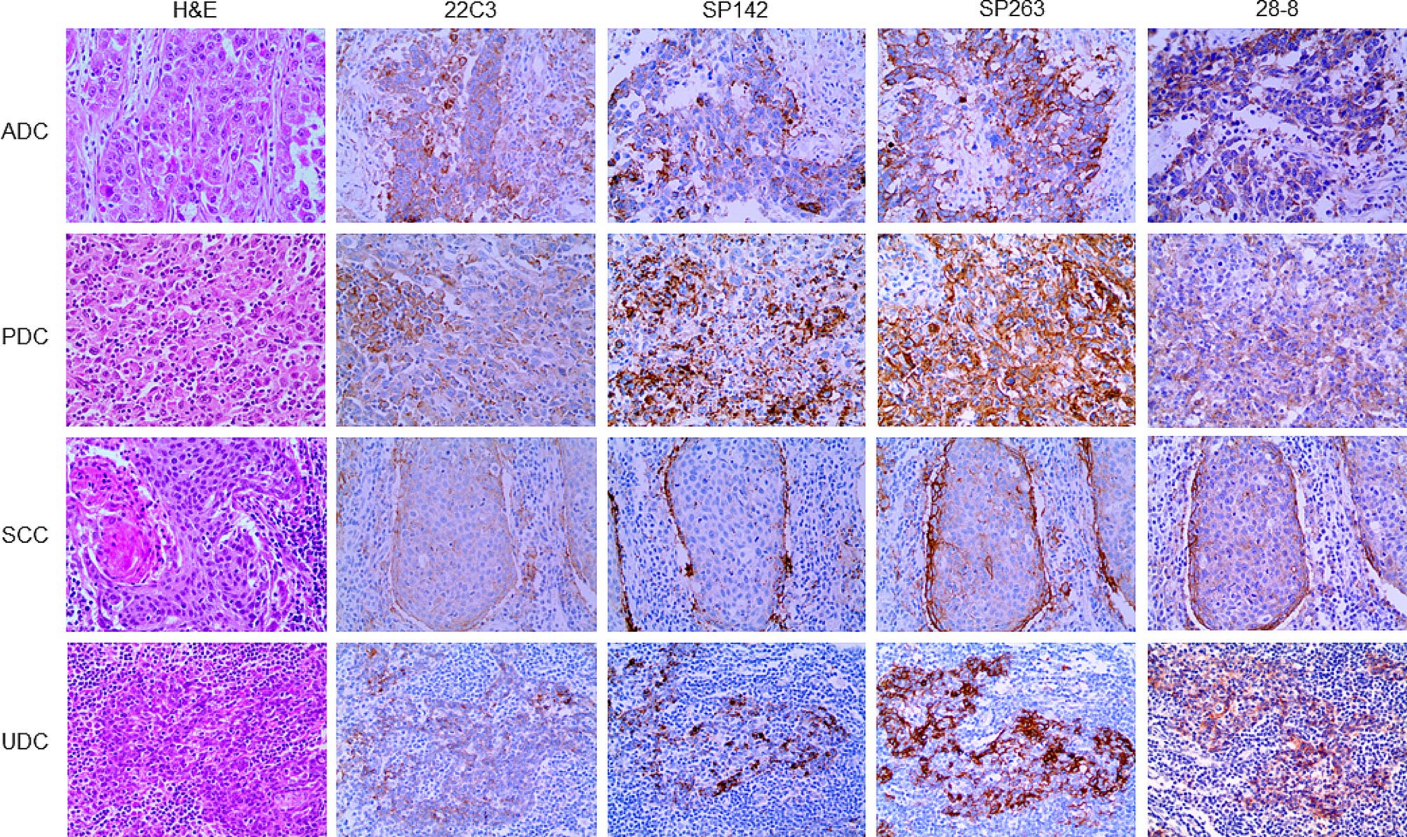
PD-L1 expression in tumor cells and immune cells in CUP histologic subtypes. In CUP, PD-L1 expression can be observed in both tumor and immune cells with varying proportions and intensities for the four histologic subtypes of ADC, PDC, SCC, and UDC using the four PD-L1 antibodies: 22C3, SP142, SP263, and 28 − 8. ADC, adenocarcinoma; CUP, carcinoma of unknown primary; PDC, poorly differentiated carcinoma; PD-L1, programmed death-ligand 1; SCC, squamous cell carcinoma; UDC, undifferentiated carcinoma

In CUP, tumor and immune cells exhibited PD-L1 expression at varying proportions and intensities (Fig. 1). PD-L1 expression was examined in CUP using cutoff values as follows: TPS ≥ 1%, CPS ≥ 10 for SP142; TC ≥ 50%, IC ≥ 10% for 22C3; TC and IC ≥ 1% for 28 − 8 and SP263. PD-L1 positivity rates ranged between 5.6 and 48.6%, with the lowest rate of 5.6% observed in PD-L1 SP142 TC and the highest rate of 48.6% in PD-L1 SP263 IC. PD-L1 positivity rates did not show significant differences according to histologic subtype (Table 1), clinical subtype (Table 2), or CK7/CK20 pattern (Table 3) across clones.

**Table 1:** PD-L1 expression in CUP according to the histologic subtype

PD-L1 status	Total *n* = 72 (%)	Histologic subtype				*p*-value
		ADC (*n* = 22) (%)	PDC (*n* = 15) (%)	SCC (*n* = 19) (%)	UDC (*n* = 16) (%)	
PD-L1 22C3 TPS						0.694
Negative (1<)	58 (80.6)	19 (86.4)	11 (73.3)	16 (84.2)	12 (75.0)	
Positive (≥ 1)	14 (19.4)	3 (13.6)	4 (26.7)	3 (15.8)	4 (25.0)	
PD-L1 22C3 CPS						0.606
Negative (10<)	60 (83.3)	20 (90.9)	12 (80.0)	16 (84.2)	12 (75.0)	
Positive (≥ 10)	12 (16.7)	2 (9.1)	3 (20.0)	3 (15.8)	4 (25.0)	
PD-L1 SP142 TC						0.423
Negative (50<)	68 (94.4)	22 (100.0)	14 (93.3)	18 (94.7)	14 (87.5)	
Positive (≥ 50)	4 (5.6)	0 (0.0)	1 (6.7)	1 (5.3)	2 (12.5)	
PD-L1 SP142 IC						0.842
Negative (10<)	66 (91.7)	20 (90.9)	13 (86.7)	18 (94.7)	15 (93.8)	
Positive (≥ 10)	6 (8.3)	2 (9.1)	2 (13.3)	1 (5.3)	1 (6.3)	
PD-L1 SP263 TC						0.361
Negative (1<)	46 (63.9)	16 (72.7)	10 (66.7)	9 (47.4)	11 (68.8)	
Positive (≥ 1)	26 (36.1)	6 (27.3)	5 (33.3)	10 (52.6)	5 (31.3)	
PD-L1 SP263 IC						0.478
Negative (1<)	37 (51.4)	14 (63.6)	6 (40.0)	10 (52.6)	7 (43.8)	
Positive (≥ 1)	35 (48.6)	8 (36.4)	9 (60.0)	9 (47.4)	9 (56.3)	
PD-L1 28 − 8 TC						0.918
Negative (1<)	56 (77.8)	18 (81.8)	12 (80.0)	14 (73.7)	12 (75.0)	
Positive (≥ 1)	16 (22.2)	4 (18.2)	3 (20.0)	5 (26.3)	4 (25.0)	
PD-L1 28 − 8 IC						0.819
Negative (1<)	53 (73.6)	17 (77.3)	12 (80.0)	13 (68.4)	11 (68.8)	
Positive (≥ 1)	19 (26.4)	5 (22.7)	3 (20.0)	6 (31.6)	5 (31.3)	

ADC, adenocarcinoma; CPS, combined positive score; CUP, carcinoma of unknown primary; IC, immune cell score; PDC, poorly differentiated carcinoma; PD-L1, programmed death-ligand 1; SCC, squamous cell carcinoma; TPS, tumor proportion score; UDC, undifferentiated carcinoma

**Table 2:** PD-L1 expression in CUP according to the clinical subtype

PD-L1 status	Total (*n* = 72) (%)	Clinical subtype		*p*-value
		Favorable type (*n* = 17) (%)	Unfavorable type (*n* = 55) (%)	
PD-L1 22C3 TPS				0.830
Negative (1<)	58 (80.6)	14 (82.4)	44 (80.0)	
Positive (≥ 1)	14 (19.4)	3 (17.6)	11 (20.0)	
PD-L1 22C3 CPS				0.901
Negative (10<)	60 (83.3)	14 (82.4)	46 (83.6)	
Positive (≥ 10)	12 (16.7)	3 (17.6)	9 (16.4)	
PD-L1 SP142 TC				0.946
Negative (50<)	68 (94.4)	16 (94.1)	52 (94.5)	
Positive (≥ 50)	4 (5.6)	1 (5.9)	3 (5.5)	
PD-L1 SP142 IC				0.558
Negative (10<)	66 (91.7)	15 (88.2)	51 (92.7)	
Positive (≥ 10)	6 (8.3)	2 (11.8)	4 (7.3)	
PD-L1 SP263 TC				0.619
Negative (1<)	46 (63.9)	10 (58.8)	36 (65.5)	
Positive (≥ 1)	26 (36.1)	7 (41.2)	19 (34.5)	
PD-L1 SP263 IC				0.683
Negative (1<)	37 (51.4)	8 (47.1)	29 (52.7)	
Positive (≥ 1)	35 (48.6)	9 (52.9)	26 (47.3)	
PD-L1 28 − 8 TC				0.882
Negative (1<)	56 (77.8)	13 (76.5)	43 (78.2)	
Positive (≥ 1)	16 (22.2)	4 (23.5)	12 (21.8)	
PD-L1 28 − 8 IC				0.341
Negative (1<)	53 (73.6)	11 (64.7)	42 (76.4)	
Positive (≥ 1)	19 (26.4)	6 (35.3)	13 (23.6)	

CPS, combined positive score CUP, carcinoma of unknown primary; IC, immune cell score; PD-L1, programmed death-ligand 1; TPS, tumor proportion score

**Table 3:** PD-L1 expression in CUP according to the CK7 and CK20 pattern

PD-L1 status	Total (*n* = 72) (%)	CK7/CK20 pattern				*p*-value
		CK7(+)/CK20(-) (*n* = 37) (%)	CK7(+)/CK20(+) (*n* = 3) (%)	CK7(-)/CK20(+) (*n* = 3) (%)	CK7(-)/CK20(-) (*n* = 29) (%)	
PD-L1 22C3 TPS						0.798
Negative (1<)	58 (80.6)	31 (83.8)	2 (66.7)	2 (66.7)	23 (79.3)	
Positive (≥ 1)	14 (19.4)	6 (16.2)	1 (33.3)	1 (33.3)	14 (19.4)	
PD-L1 22C3 CPS						0.750
Negative (10<)	60 (83.3)	31 (83.8)	2 (66.7)	3 (100.0)	24 (82.8)	
Positive (≥ 10)	12 (16.7)	6 (16.2)	1 (33.3)	0 (0.0)	5 (17.2)	
PD-L1 SP142 TC						0.154
Negative (50<)	68 (94.4)	36 (97.3)	2 (66.7)	3 (100.0)	27 (93.1)	
Positive (≥ 50)	4 (5.6)	1 (2.7)	1 (33.3)	0 (0.0)	2 (6.9)	
PD-L1 SP142 IC						0.820
Negative (10<)	66 (91.7)	33 (89.2)	3 (100.0)	3 (100.0)	27 (93.1)	
Positive (≥ 10)	6 (8.3)	4 (10.8)	0 (0.0)	0 (0.0)	2 (6.9)	
PD-L1 SP263 TC						0.527
Negative (1<)	46 (63.9)	26 (70.3)	1 (33.3)	2 (66.7)	17 (58.6)	
Positive (≥ 1)	26 (36.1)	11 (29.7)	2 (66.7)	1 (33.3)	12 (41.4)	
PD-L1 SP263 IC						0.880
Negative (1<)	37 (51.4)	19 (51.4)	2 (66.7)	2 (66.7)	14 (48.3)	
Positive (≥ 1)	35 (48.6)	18 (48.6)	1 (33.3)	1 (33.3)	15 (51.7)	
PD-L1 28 − 8 TC						0.868
Negative (1<)	56 (77.8)	30 (81.1)	2 (66.7)	2 (66.7)	22 (75.9)	
Positive (≥ 1)	16 (22.2)	7 (18.9)	1 (33.3)	1 (33.3)	7 (24.1)	
PD-L1 28 − 8 IC						0.838
Negative (1<)	53 (73.6)	26 (70.3)	2 (66.7)	2 (66.7)	23 (79.3)	
Positive (≥ 1)	19 (26.4)	11 (29.7)	1 (33.3)	1 (33.3)	6 (20.7)	

CPS, combined positive score CUP, carcinoma of unknown primary; IC, immune cell score; PD-L1, programmed death-ligand 1; TPS, tumor proportion score

### Difference and concordance of PD-L1 expression in CUP according to PD-L1 antibody clones and Scoring systems

We then analyzed differences in PD-L1 expression among the four clones and scoring systems in CUP. For the TC system, PD-L1 positivity ranged between 18.1 and 36.1% for a cutoff value of 1% and between 4.2 and 20.8% for a cutoff value of 50%. Among the examined clones, 22C3 and SP263 showed the lowest and highest positivity rates, respectively. For the IC system, PD-L1 positivity ranged between 26.4 and 48.6% for a cutoff value of 1% and between 9.7 and 38.9% for a cutoff value of 10%. Among the clones, 28 − 8 and SP263 exhibited the lowest and highest positivity rates, respectively (Table 4).

**Table 4:** PD-L1 positivity for TC and IC in CUP according to PD-L1 clones

PD-L1 clone	TC ≥ 1%, *n* (%)	TC ≥ 50%, *n* (%)	IC ≥ 1%, *n* (%)	IC ≥ 10%, *n* (%)
22C3	14 (19.4)	3 (4.2)	24 (33.3)	8 (11.1)
28 − 8	16 (22.2)	10 (13.9)	19 (26.4)	7 (9.7)
SP142	13 (18.1)	4 (5.6)	30 (41.7)	8 (11.1)
SP263	26 (36.1)	15 (20.8)	35 (48.6)	28 (38.9)

CPS, combined positive score CUP, carcinoma of unknown primary; IC, immune cell score; PD-L1, programmed death-ligand 1; TPS, tumor proportion score

Next, we examined the concordance of PD-L1 expression among clones according to the scoring system (Table 5). For TC ≥ 1%, all clones showed moderate or high agreement, with the highest agreement between 22C3 and SP142 (OA = 93.1%, ≥ =0.772) and the lowest agreement between 22C3 and SP263 (OA = 83.3%, ≥ =0.599). For TC κ 50%, all clones showed fair or higher agreement, with the highest agreement between 28 − 8 and SP263 (OA = 90.3%, κ = 0.664) and the lowest agreement between 22C3 and SP263 (OA = 83.3%, κ = 0.284). For IC ≥ 1%, all clones showed moderate or higher agreement, with the highest agreement between SP263 and SP142 (OA = 90.3%, κ = 0.805) and the lowest agreement between 28 − 8 and SP263 (OA = 77.8%, κ = 0.550). For IC κ 10%, all clones showed fair or high agreement, with the highest agreement between 22C3 and SP142 (OA = 91.7%, κ = 0.578) and the lowest agreement between SP142 and SP263 (OA = 69.4%, κ = 0.261).

**Table 5:** Pairwise comparisons for concordance and kappa statistics among PD-L1 clones according to the scoring system

Scoring system and PD-L1 clone pair	Overall agreement (OA) (%)	Kappa coefficient (95%CI)	Category of agreement
TC ≥ 1%			
22C3 vs. 28 − 8	66 (91.7)	0.748 (0.651–0.845)	Substantial
22C3 vs. SP142	67 (93.1)	0.772 (0.675–0.869)	Substantial
22C3 vs. SP263	60 (83.3)	0.599 (0.502–0.696)	Moderate
28 − 8 vs. SP142	61 (84.7)	0.526 (0.402–0.650)	Moderate
28 − 8 vs. SP263	60 (83.3)	0.606 (0.508–0.704)	Moderate
SP142 vs. SP263	57 (79.2)	0.493 (0.389–0.597)	Moderate
TC ≥ 50%			
22C3 vs. 28 − 8	65 (90.3)	0.425 (0.256–0.594)	Moderate
22C3 vs. SP142	67 (93.1)	0.250 (0.020–0.480)	Fair
22C3 vs. SP263	60 (83.3)	0.284 (0.152–0.416)	Fair
28 − 8 vs. SP142	66 (91.7)	0.534 (0.373–0.695)	Moderate
28 − 8 vs. SP263	65 (90.3)	0.664 (0.548–0.780)	Substantial
SP142 vs. SP263	61 (84.7)	0.365 (0.229–0.501)	Fair
IC ≥ 1%			
22C3 vs. 28 − 8	65 (90.3)	0.769 (0.687–0.851)	Substantial
22C3 vs. SP142	64 (88.9)	0.765 (0.688–0.842)	Substantial
22C3 vs. SP263	59 (81.9)	0.636 (0.549–0.723)	Substantial
28 − 8 vs. SP142	59 (81.9)	0.608 (0.515–0.701)	Moderate
28 − 8 vs. SP263	56 (77.8)	0.550 (0.461–0.639)	Moderate
SP142 vs. SP263	65 (90.3)	0.805 (0.736–0.874)	Substantial
IC ≥ 10%			
22C3 vs. 28 − 8	65 (90.3)	0.479 (0.311–0.647)	Moderate
22C3 vs. SP142	66 (91.7)	0.578 (0.423–0.733)	Moderate
22C3 vs. SP263	52 (72.2)	0.328 (0.233–0.423)	Fair
28 − 8 vs. SP142	63 (87.5)	0.331(0.159–0.503)	Fair
28 − 8 vs. SP263	51 (70.8)	0.289(0.197–0.381)	Fair
SP142 vs. SP263	50 (69.4)	0.261(0.166–0.356)	Fair

CI, confidence interval; CPS, combined positive score; CUP, carcinoma of unknown primary; IC, immune cell score; PD-L1, programmed death-ligand 1; TPS, tumor proportion score

### Impact of clinicopathologic factors and PD-L1 status on prognosis of CUP

**Fig. 2:**
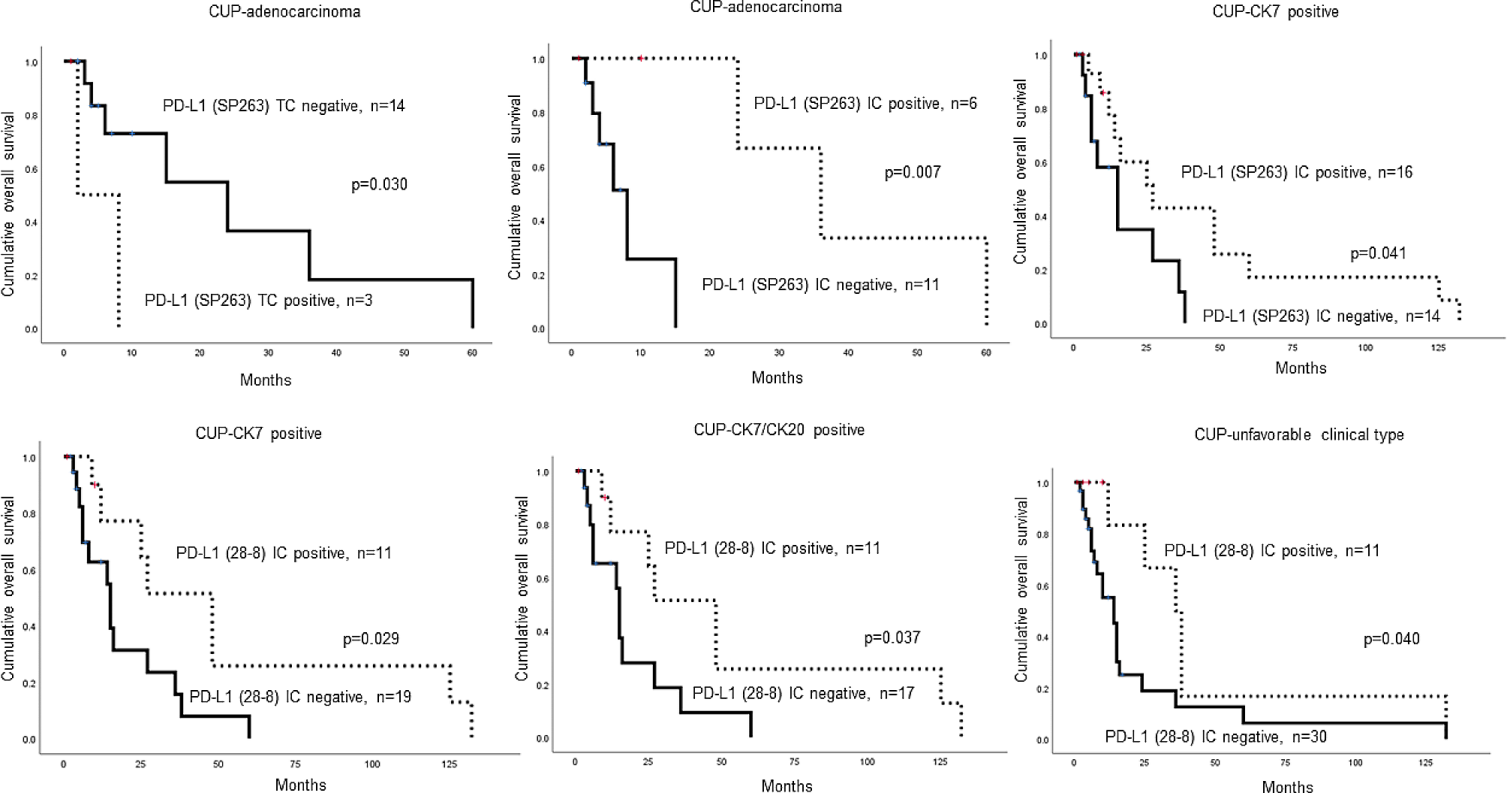
Impact of clinicopathologic factors and PD-L1 status on the prognosis of CUP. In the case of ADC, PD-L1 SP263 TC positivity (*p* = 0.030) and PD-L1 SP263 IC negativity (*p* = 0.007) show a significant association with shorter overall survival. In CK7 positive CUP, PD-L1 SP263 IC negativity (*p* = 0.041) and PD-L1 28 − 8 IC negativity (*p* = 0.029) are significantly associated with shorter overall survival, while in CK7 and CK20 positive CUP and unfavorable clinical type CUP, PD-L1 28 − 8 IC negativity (*p* = 0.037 and *p* = 0.040, respectively) shows a significant association with shorter overall survival. ADC, adenocarcinoma; CUP, carcinoma of unknown primary; IC, immune cell score; PDC, poorly differentiated carcinoma; PD-L1, programmed death-ligand 1; SCC, squamous cell carcinoma; UDC, undifferentiated carcinoma

We subsequently performed univariate analysis to determine the impact of clinicopathological factors and PD-L1 expression on prognosis. We observed that the histological subtype was associated with shorter overall survival (OS) (UDC > SCC > ADC > PDC, *p* = 0.030), whereas PD-L1 expression was not significantly associated with shorter OS (Table 6). In subgroup analysis, PD-L1 SP263 TC positivity (*p* = 0.030) and PD-L1 SP263 IC negativity (*p* = 0.007) were significantly associated with shorter OS for CUP with ADC histologic subtypes. For CK7 positive CUP, PD-L1 SP263 IC negativity (*p* = 0.041) and PD-L1 28 − 8 IC negativity (*p* = 0.029) were significantly associated with a shorter OS. For CK7 and CK20 positive CUP and unfavorable clinical type CUP, PD-L1 28 − 8 IC negativity (*p* = 0.037 and *p* = 0.040, respectively) was significantly associated with shorter OS (Fig. 2).

**Table 6:** The impact of clinicopathologic and PD-L1 parameters on time to survival by univariate analysis

Parameters	No. of patients (*n* = 51^*^) (%)			Overall survival	
	No. of cases	Patient death		Median survival (95% CI) (months)	*p*-value
Sex					0.267
Male	32	24		33 (21–46)	
Female	19	14		25 (8–41)	
Histologic subtype					**0.030**
ADC	17	9		22 (8–35)	
PDC	14	13		18 (12–25)	
SCC	16	11		32 (18–47)	
UDC	9	8		64 (24–104)	
Clinical subtype					0.239
Favorable type	15	14		41 (23–58)	
Unfavorable type	41	27		28 (14–42)	
CK7					0.892
Negative	26	19		32 (17–47)	
Positive	30	22		32 (17–48)	
CK20					0.386
Negative	52	37		33 (22–45)	
Positive	4	4		21 (2–39)	
CK7/CK20 pattern					0.804
CK7 (+)/CK20 (-)	28	20		33 (17–50)	
CK7 (+)/CK20 (+)	2	2		23 (0–52)	
CK7(-)/CK20(+)	2	2		19 (0–52)	
CK7(-)/CK20(-)	24	17		33 (17–50)	
PD-L1 22C3 TPS					0.917
Negative (1<)	45	31		32 (20–44)	
Positive (≥ 1)	11	10		32 (9–56)	
PD-L1 22C3 CPS					0.482
Negative (10<)	47	33		31 (19–43)	
Positive (≥ 10)	9	8		39 (11–67)	
PD-L1 SP142 TC					0.760
Negative (50<)	53	38		32 (21–44)	
Positive (≥ 50)	3	3		33 (25–42)	
PD-L1 SP142 IC					0.907
Negative (10<)	52	37		33 (21–44)	
Positive (≥ 10)	4	4		29 (0–58)	
PD-L1 SP263 TC					0.868
Negative (1<)	35	24		33 (19–47)	
Positive (≥ 1)	21	17		31 (14–47)	
PD-L1 SP263 IC					0.095
Negative (1<)	27	18		22 (12–32)	
Positive (≥ 1)	29	23		40 (23–56)	
PD-L1 28 − 8 TC					0.472
Negative (1<)	44	32		31 (19–43)	
Positive (≥ 1)	12	9		38 (13–63)	
PD-L1 28 − 8 IC					0.104
Negative (1<)	39	29		26 (15–38)	
Positive (≥ 1)	17	12		46 (23–69)	

ADC, adenocarcinoma; CI, confidence interval; CPS, combined positive score; CUP, carcinoma of unknown primary; IC, immune cell score; PDC, poorly differentiated carcinoma; PD-L1, programmed death-ligand 1; SCC, squamous cell carcinoma; TPS, tumor proportion score; UDC, undifferentiated carcinoma. ^*^ Of the 72 patients, clinical follow-up data were available for 51

## Discussion

In the present study, we determined the expression of PD-L1 in various CUP clones, detecting various positive rates depending on the antibodies used, the applied scoring system, and cutoff values. Although PD-L1 expression in CUP remains poorly established, a positivity rate of 22% in tumor cells has been reported [40] using the antibody SP142, with the positivity criteria defined as moderate (2+) membranous positivity in at least 5% of tumor cells, indicating that the previous criteria were TC ≥ 5%. In addition, PD-L1 was found to be expressed in tumor cells in 14% of the CUP cases [41]; the antibody used was 22C3, and the positivity criteria were defined as at least 50% of tumor cells being positive, indicating that the previous criteria were TPS ≥ 50%. In the current study, the positivity rates were 18.1% (for TC ≥ 1%) and 5.6% (for TC ≥ 50%) using SP142, and 19.4% (for TC ≥ 1%) and 4.2% (for TC ≥ 50%) with 22C3. The PD-L1 positivity rate varied depending on the PD-L1 antibody clone, scoring system, and cutoff values, as well as based on the interpretation by the pathologist and sample tissue type. Therefore, a direct comparison can be challenging. Although some studies have examined the expression of PD-L1 using only one PD-L1 antibody, no study has explored PD-L1 expression using multiple PD-L1 antibodies with various scoring systems or cutoff values. As previously mentioned, various factors can impact the results of PD-L1 IHC in tumors, including the PD-L1 antibody clone, scoring system, cutoff value, interpretation pathologist, sample tissue type (biopsy or resection), and primary or metastasis. Accordingly, several studies have investigated the expression and consistency of PD-L1 according to these factors in various types of cancers. PD-L1 expression has been extensively explored in cancers such as non-small cell lung carcinoma (NSCLC), TNBC, melanoma, renal cell carcinoma, bladder cancer, and gastric cancer. The positivity rates of PD-L1 in each cancer type were as follows: NSCLC (TC = 23–86%, IC = 23–68%) [42], breast TNBC (IC = 23–74%, CPS = 17–81%) [43], renal cell carcinoma (TPS = 25–60%) [44], bladder cancer (TC = 12–72%) [45], and gastric cancer (TC = 15–69%) [23].

In tumors, the main function of PD-L1 is to predict the response to immune checkpoint inhibitors (ICI), and various clinical trials are underway to optimize its function as a predictive factor, depending on the type of tumor. Accordingly, a companion diagnosis has been established in clinical practice for each cancer type, determining the optimal PD-L1 antibody clone, IHC platform, scoring system, cutoff value, and specific ICI. Representative tumors include NSCLC, TNBC, urothelial carcinoma, uterine cervical cancer, and gastric/esophageal cancer. Therefore, additional preclinical and clinical studies are required to determine the optimal PD-L1 conditions for CUP. Although the possibility of an ICI therapy response according to PD-L1 expression status in CUP warrants clinical trials and extensive research, a potential response to ICI therapy according to the PD-L1 expression status can be sufficiently suggested.

Currently, the treatment approach in CUP involves site-specific therapy if the tissue-of-origin is determined using an IHC panel and/or molecular tissue-of-origin assay [7–9, 30]. Given that the efficacy of ICI therapy based on PD-L1 has been confirmed in NSCLC, TNBC, urothelial carcinoma, uterine cervical cancer, and gastric/esophageal cancer, if the tissue origin is determined for CUP using an IHC panel and/or molecular tissue-of-origin assay, ICI therapy could be initiated on assessing PD-L1 expression. However, it is necessary to consider that the currently defined PD-L1 clones, IHC platforms, scoring systems, and cutoff values for each cancer type tend to differ; therefore, additional research is needed to determine whether different PD-L1 evaluation systems should be used according to the tissue origin in CUP.

Based on the subgroup analysis of CUP, PD-L1 SP263 TC positivity, PD-L1 SP263 IC negativity, and PD-L1 28 − 8 IC negativity were associated with a poor prognosis. Other tumors, including urothelial carcinoma, NSCLC, head and neck cancer, and liver cholangiocarcinoma, have shown similar results, where PD-L1 expression in tumor cells was associated with poor prognosis, whereas PD-L1 expression in immune cells was associated with better prognosis [46–50].

In this study, only PD-L1 staining was conducted. However, previous studies in other cancer types have performed double staining such as CD68/PD-L1 to distinguish staining differences between PD-L1 and tumor-associated macrophages (TAMs) and other immune cells, and have presented differences in tumor subtypes and prognosis accordingly [51, 52]. Therefore, dual staining like CD68/PD-L1 can provide important insights into the role of immune cells in the tumor microenvironment and the mechanisms of tumor immune evasion. This could aid in developing treatment strategies and identifying the origin of tumors. Therefore, additional research on dual staining, such as CD68/PD-L1, is deemed necessary to accurately characterize the tumor properties and develop personalized treatment strategies, especially in cases like CUP where the tumor origin is unknown.

One limitation of this study is that PD-L1 staining was conducted on a limited amount of tissue using TMA, which may not adequately reflect tumor heterogeneity. Previous studies investigating the differences in PD-L1 expression between biopsy and surgical tissue in various cancer types have shown a concordance rate of 70% or higher in most cases [53–56]. Additionally, in clinical practice, obtaining small biopsies rather than excising the entire lesion surgically is more common in cases of CUP, suggesting that the results from TMA studies may be more similar to the actual clinical environment. Moreover, in cases where small biopsies are not feasible due to various clinical circumstances, cytological samples may be considered for assessing PD-L1 status in CUP patients. Previous studies have reported moderate or higher concordance rates between cytology and histology samples regarding PD-L1 expression [57–59], indicating the need for additional research on PD-L1 expression in cytological samples from CUP patients.

## Conclusions

In conclusion, PD-L1 expression was observed in CUP, with varying positivity rates depending on the antibody and scoring system employed. There was no difference in PD-L1 expression based on histological or clinical subtypes. Therefore, ICI treatment based on PD-L1 expression in CUP can be an effective treatment strategy.

### Electronic supplementary material

Below is the link to the electronic supplementary material.

Supplementary Material 1

## Data Availability

All data supporting the findings of this study are available within the paper and its Supplementary Information.
