# Supplementary material for: Programmed death-ligand 1 expression in carcinoma of unknown primary
Source: BMC Cancer. 2024 Jun 6;24:689. doi: 10.1186/s12885-024-12437-w (PMC11155179; doi:10.1186/s12885-024-12437-w)
Supplement: Supplementary file 1 — Supplementary Material 1 [file 12885_2024_12437_MOESM1_ESM.docx]

Supplementary Table 1. Clone, dilution, and source of antibodies used

| Antibody | Clone | Dilution | Source |
| --- | --- | --- | --- |
| PD-L1 related | | | |
| PD-L1 22C3 | 22C3 | N/A | DAKO |
| PD-L1 28-8 | 28-8 | N/A | DAKO |
| PD-L1 SP142 | SP142 | N/A | Ventana Medical Systems |
| PD-L1 SP 263 | SP263 | N/A | Ventana Medical Systems |
| CK related | | | |
| CK7 | EPR17078 | 1:100 | Abcam |
| CK20 | EPR1622Y | 1:100 | Abcam |

Supplementary Table 2. Clinicopathologic characteristics of patients according to the histologic subtype

| Clinical parameters | Total  N= 72  (%) | Histologic subtype | | | | p-value |
| --- | --- | --- | --- | --- | --- | --- |
|  |  | ADC (n=22) (%) | PDC (n=15) (%) | SCC (n=19) (%) | UDC (n=16) (%) |  |
| Age  (years, mean±SD) | 54.8±11.8 | 59.3±12.5 | 53.6±12.6 | 56.6±8.6 | 47.3±10.2 | **0.013** |
| Sex |  |  |  |  |  | 0.617 |
| Female | 24 (33.3) | 9 (40.9) | 6 (40.0) | 5 (26.3) | 4 (25.0) |  |
| Male | 48 (66.7) | 13 (59.1) | 9 (60.0) | 14 (73.7) | 12 (75.0) |  |
| Clinical subtype |  |  |  |  |  | **0.003** |
| Favorable type | 17 (23.6) | 2 (9.1) | 4 (26.7) | 10 (52.6) | 1 (6.3) |  |
| Unfavorable type | 55 (76.4) | 20 (90.9) | 11 (73.3) | 9 (47.4) | 15 (93.8) |  |
| Organs involved |  |  |  |  |  | 0.160 |
| Lymph node | 49 (68.1) | 10 (45.5) | 11 (73.3) | 18 (94.7) | 10 (62.5) |  |
| Bone | 8 (11.1) | 5 (22.7) | 1 (6.7) | 0 (0.0) | 2 (12.5) |  |
| Brain | 7 (9.7) | 3 (13.6) | 1 (6.7) | 1 (5.3) | 2 (12.5) |  |
| Other | 8 (11.1) | 4 (18.2) | 2 (13.3) | 0 (0.0) | 2 (12.5) |  |
| Postoperative treatment |  |  |  |  |  | **0.007** |
| None | 18 (25.0) | 6 (27.3) | 2 (13.3) | 4 (21.1) | 6 (37.5) |  |
| Chemotherapy | 25 (34.7) | 11 (50.0) | 5 (33.3) | 3 (15.8) | 6 (37.5) |  |
| Radiation therapy | 12 (16.7) | 5 (22.7) | 0 (0.0) | 5 (26.3) | 2 (12.5) |  |
| Chemo-radiation therapy | 17 (23.6) | 0 (0.0) | 8 (53.3) | 7 (36.8) | 2 (12.5) |  |
| CK7 |  |  |  |  |  | 0.372 |
| Negative | 32 (44.4) | 7 (31.8) | 9 (60.0) | 8 (42.1) | 8 (50.0) |  |
| Positive | 40 (55.6) | 15 (68.2) | 6 (40.0) | 11 (57.9) | 8 (50.0) |  |
| CK20 |  |  |  |  |  | 0.428 |
| Negative | 66 (91.7) | 19 (86.4) | 15 (100.0) | 18 (94.7) | 14 (87.5) |  |
| Positive | 6 (8.3) | 3 (13.6) | 0 (0.0) | 1 (5.3) | 2 (12.5) |  |
| CK7/CK20 pattern |  |  |  |  |  | 0.522 |
| CK7 (+)/CK20 (-) | 37 (51.4) | 14 (63.6) | 6 (40.0) | 10 (52.6) | 7 (43.8) |  |
| CK7 (+)/CK20 (+) | 3 (4.2) | 1 (4.5) | 0 (0.0) | 1 (5.3) | 1 (6.3) |  |
| CK7(-)/CK20(+) | 3 (4.2) | 2 (9.1) | 0 (0.0) | 0 (0.0) | 1 (6.3) |  |
| CK7(-)/CK20(-) | 29 (40.3) | 5 (22.7) | 9 (60.0) | 8 (42.1) | 7 (43.8) |  |

PD: poorly differentiated carcinoma, AD: adenocarcinoma, SQ: squamous cell carcinoma, UD: undifferentiated carcinoma

| Supplementary Table 3. Clinicopathologic characteristics of patients according to the clinical subtype | | | | |
| --- | --- | --- | --- | --- |
| Clinical parameters | Total  (n=72)  (%) | clinical subtype | | p-value |
|  |  | Favorable type  (n=17) (%) | Unfavorable type  (n=55) (%) |  |
| Age  (years, mean±SD) | 54.8±11.8 | 50.4±8.1 | 56.1±12.4 | 0.084 |
| Sex |  |  |  | 0.844 |
| Female | 24 (33.3) | 6 (35.3) | 18 (32.7) |  |
| Male | 48 (66.7) | 11 (64.7) | 37 (67.3) |  |
| Organs involved |  |  |  | 0.336 |
| Lymph node | 49 (68.1) | 14 (82.4) | 35 (63.6) |  |
| Bone | 8 (11.1) | 2 (11.8) | 6 (10.9) |  |
| Brain | 7 (9.7) | 0 (0.0) | 7 (12.7) |  |
| Other | 8 (11.1) | 1 (5.9) | 7 (12.7) |  |
| Postoperative treatment |  |  |  | 0.329 |
| None | 18 (25.0) | 6 (35.3) | 12 (21.8) |  |
| Chemotherapy | 25 (34.7) | 3 (17.6) | 22 (40.0) |  |
| Radiation therapy | 12 (16.7) | 4 (23.5) | 8 (14.5) |  |
| Chemo-radiation therapy | 17 (23.6) | 4 (23.5) | 13 (23.6) |  |
| CK7 |  |  |  |  |
| Negative | 32 (44.4) | 5 (29.4) | 27 (49.1) |  |
| Positive | 40 (55.6) | 12 (70.6) | 28 (50.9) |  |
| CK20 |  |  |  | 0.676 |
| Negative | 66 (91.7) | 16 (94.1) | 50 (90.9) |  |
| Positive | 6 (8.3) | 1 (5.9) | 5 (9.1) |  |
| CK7/CK20 pattern |  |  |  | 0.474 |
| CK7 (+)/CK20 (-) | 37 (51.4) | 11 (64.7) | 26 (47.3) |  |
| CK7 (+)/CK20 (+) | 3 (4.2) | 1 (5.9) | 2 (3.6) |  |
| CK7(-)/CK20(+) | 3 (4.2) | 0 (0.0) | 3 (5.5) |  |
| CK7(-)/CK20(-) | 29 (40.3) | 5 (29.4) | 24 (43.6) |  |
